# Feasibility and reliability of pressure algometry for mechanical nociceptive threshold quantification in lambs in a field environment

**DOI:** 10.3389/fpain.2026.1696631

**Published:** 2026-06-24

**Authors:** Charlotte Helen Johnston, Danila Marini, Stefan T. Musolino, Daniel T. Barratt, Mark R. Hutchinson

**Affiliations:** 1 School of Biomedicine, Faculty of Health and Medical Sciences, University of Adelaide, Australia, Adelaide, SA, Australia; 2 Davies Livestock Research Centre, University of Adelaide, Roseworthy, SA, Australia; 3 School of Animal and Veterinary Sciences, University of Adelaide, Roseworthy, SA, Australia; 4 Australian Research Council Centre of Excellence for Nanoscale BioPhotonics, University of Adelaide, Adelaide, SA, Australia

**Keywords:** agreement, algometer, intra-operator reliability, mechanical nociceptive threshold, precision, sheep, tail docking

## Abstract

**Objective:**

To validate a modified algometer method for assessing mechanical nociceptive threshold (MNT) in lambs before and after husbandry procedures (marking) under field conditions by testing the precision, intra-operator reliability and agreement, and minimal detectable change (MDC).

**Animals:**

Forty-six randomly allocated Merino lambs: 6 control (sham handled), 13 hot knife tail docked, 13 rubber ring tail docked, 7 hot knife tail docked and rubber ring castrated, and 7 rubber ring tail docked and castrated.

**Methods:**

MNT (mean triplicate measurements at each replicate) was assessed at the tail base, using three replicate measurements before, and three hours after marking to assess precision, reliability, agreement, and MDC. A single operator performed all testing, with lambs restrained by one of three handlers (randomly) for each replicate. Within-animal/-occasion (pre- or post-marking) relative and absolute precision were evaluated using the coefficient of variation (CV) and a combination of standard deviation (SD) and dispersion modelling, respectively. Agreement was assessed using Bland-Altman plots, and linear mixed effects regression was used to determine the intra-class correlation coefficient (ICC) for intra-operator reliability, and any systematic effects of replicate order or handler. MDC was calculated from the standard error of measurement. Responsiveness to marking was analyzed by comparing MNT change from pre- to post-marking using linear mixed effects regression.

**Results:**

The overall median MNT was 3.07 kgf (IQR = 2.00–3.84, range = 1.23–5.00). Greater body weight was associated with significantly higher absolute imprecision (*p* = 7 × 10^−5^). Median CV was 12.5% (IQR = 7.29–18.16, range = 1.5–55.4) and was significantly higher post-marking (*p* = 0.0003), likely related to lower post-marking MNT. The agreement between replicates was good with small mean differences (0.02–0.26 kgf), although there were wide Bland-Altman limits of agreement (2.27–3.51 kgf). There was good intra-operator reliability [ICC (95% CI) = 0.83 (0.81–0.94)]. The minimum detectable change was 1.38 kgf. There was a significant reduction in MNT after marking for all marking groups (*p* < 0.01).

**Conclusions:**

Modified algometer MNT measurement in lambs around the time of marking was feasible with sufficient precision and responsiveness to demonstrate a reduction in nociceptive threshold following husbandry procedures.

## 1 Introduction

Objective diagnosis and quantification of pain in animals remains a major challenge to the advancement of farm animal welfare and the development of effective pain relief (1–4). Painful husbandry procedures are routinely performed on young livestock with uptake of analgesia or anesthesia varying by region and context (5–7). Globally, some livestock producers perceive that analgesia is not necessary for procedures such as tail docking (amputation of part of the tail) and castration (removal of the testicles), largely due to the absence of overt or easily identifiable pain behaviors in these species (6, 8, 9). Whilst pain is ultimately a subjective experience, there is a need for objective and quantitative measures of nociception/pain in animals unable or inclined not to communicate their subjective experience of pain. The development of accurate and reliable methods for assessing nociception and pain will improve our ability to diagnose pain and aid in the wider implementation of effective pain mitigation strategies.

Tissue injury causes pain and evokes peripheral and central sensitization (10). Alterations in sensitivity, such as a reduction in nociceptive threshold (hyperalgesia or allodynia), indicate the presence and degree of somatosensory response to the injury. Mechanical nociceptive threshold (MNT) testing measures an individual's response to an externally applied mechanical stimulus and has been used extensively in preclinical and clinical research to investigate pain mechanisms and support the development of new analgesics. While nociceptive threshold testing is typically performed in controlled laboratory environments to reduce external variables, a range of factors can influence the results (11, 12), including the presence of conspecifics, gender of the investigator (13), age and sex of the subject (14, 15), anatomical testing site (10, 16), application rate of the stimulus (17), and prior habituation (18). This variability has been noted across species, including livestock such as cattle (18), donkeys (17), pigs (19), and sheep (20).

More recently, nociceptive threshold testing methods have been adapted to assess pain following husbandry procedures in a variety of livestock species, including cattle (21–24), pigs (25, 26), deer (27), and sheep (28–32). Often these studies are performed in field conditions where animals may be subject to a period of separation from conspecifics, restraint, and close proximity to humans, all in a novel environment. The introduction of these additional stressors, which can be more easily controlled in preclinical and clinical research, may affect pain sensitivity and influence test outcomes (33).

The validity of these techniques outside of a strictly controlled laboratory environment, and in more diverse populations of animals compared to the typical laboratory rodent study cohort, should be considered prior to utilizing such tests in field settings to ensure the experimental data can be correctly interpreted. Additionally, it is important to consider the ethical elements of using these nociceptive tests in animals, where a stimulus is applied to the point of response, usually indicating that the animal finds the stimulus aversive. If the data obtained is unreliable, then it is not ethically justified to continue using that test, and it should be refined or replaced. As such, testing the reliability of these measurement techniques within a specific experimental environment that is relevant for the intended context of use is essential for collecting robust data.

Collectively therefore, our aim was to determine the feasibility, intra-operator reliability and agreement, and responsiveness of a handheld algometer to measure mechanical nociceptive thresholds in lambs in a practically relevant environment that differs from the typical controlled laboratory environment. Specifically, we evaluated this method in lambs before and after marking, which involved vaccination and tail docking with or without castration. Findings from this study will aid in guiding the extension of nociceptive threshold testing in livestock across various environments relevant to production and advancing our understanding of pain responses in animals.

## 2 Methods

### 2.1 Ethics

All animals and procedures were approved by the University of Adelaide Animal Ethics Committee (S-2021-043). Lambs used in this study were identified and paired with their ewe at birth under ethics approval S-2024-032. All procedures were conducted in accordance with the Australian Code for the Care and Use of Animals for Scientific Purposes (2013).

### 2.2 Animals

The study included forty-six Merino lambs (*Ovis aries*), born May – June 2024, from the University of Adelaide, Roseworthy Campus flock. All animals were handled once within 12–24 h after birth to collect body weight and frame measurements and to apply an ear tag for identification. The experiment was conducted in July 2024, at the Teaching Yards on the Roseworthy Campus of The University of Adelaide, Australia. At the time of the experiment lambs were a mean (± SD) age of 37 (± 7) days (range 23–52 days) and mean (± SD) body weight of 11.34 (± 3.24) kg (range 6.5–18.6 kg).

### 2.3 Experimental design

Lambs and their mothers were walked from their home paddock to the yards on the morning of the experiment between 8 and 9am. Each lamb was briefly separated from its mother for baseline mechanical nociceptive threshold (MNT) testing (see below), which was repeated three times, with at least 1 min [median (IQR) = 3(2–4) minutes, range = 1–12 min] between tests.

After baseline testing each lamb was randomly allocated to one of five marking groups. Group assignment was randomised using MS Excel random number function with block assignment by group and sex to ensure adequate group numbers. Sample size for each group was selected balancing power to detect at least “good” reliability (intra-operator reliability intra-class correlation coefficient (ICC) ≥0.75) for a method with reliability acceptable for our context of use (ICC ∼0.75–0.85), weighting towards docked and castrated groups to match the most likely contexts of use and create relevant variability, and minimizing total animal numbers (principle of reduction). The selected sample sizes for each marking group that fit these criteria were: tail dock with hot knife (males *n* = 6, females *n* = 7), tail dock with rubber ring (males *n* = 6, females *n* = 7), tail dock with hot knife and castrated with rubber ring (males *n* = 7), tail dock and castrated with rubber ring (males *n* = 7), and sham handled control (males *n* = 3, females *n* = 3). This sample size had 80% power to show an intra-operator reliability ICC significantly (alpha = 0.05) greater than 0.75 for a “true” ICC of 0.84 (*calculateIccSampleSize* function of the *ICC.Sample.Size* package (34, 35) (treating lambs pre- and post-procedure as different animals due to expected substantial influence of stress and tissue injury on the somatosensory system).

Following group allocation, the lambs were moved to the marking area together with the ewes and placed one at a time into a marking cradle in random order. In the cradle, lambs were administered Glanvac 6 (Zoetis, Australia) subcutaneously to vaccinate against Caseous Lymphadenitis and the five main clostridial diseases: black disease, black leg, enterotoxaemia, tetanus, and malignant oedema. Each lamb was then either sham handled, tail docked, or tail docked and castrated by the same experienced operator (DM) according to their assigned marking group, as follows. In the control group, the tail and scrotum (if applicable) were handled in a similar fashion to the normal docking or castration procedure, but the actual procedure was not performed. This sham handling took the same amount of time as a routine docking and castration procedure. Tail docking and castration procedures were performed according to the Australian Animal Welfare Standards and Guidelines for Sheep (36). Rubber ring tail docking involved the application of an elastrator rubber ring (Heiniger Australia, Bibra Lake Western Australia) to the third palpable coccygeal joint from the base of the tail (37). Rubber ring castration docking involved the application of an elastrator rubber ring to the neck of the scrotum proximal to the testes. Hot knife tail docking used a gas-heated tail-docking knife (Tradeflame Lamb De-Tailer BJ5000, Australia) that was preheated and used to cut the tail at the third palpable joint and held in place for roughly two seconds to cauterize the coccygeal blood vessels.

After marking, lambs were left with their mothers for three hours before being weighed and repeating the MNT testing protocol.

### 2.4 Mechanical nociceptive threshold testing

**Figure 1:**
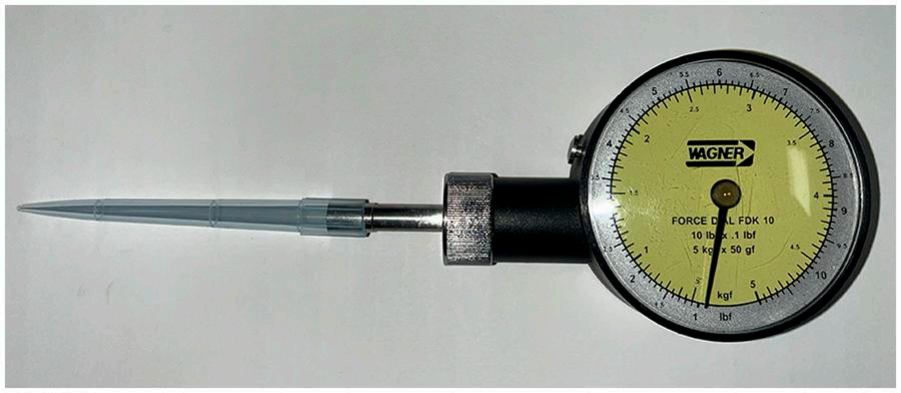
The modified algometer used in this study. A handheld algometer (Wagner Force Dial^TM^ FDK Series Push Pull Force Gage, FDK 10; Wagner Instruments, CT, USA, Maximum force 5 kgf) with a modified 1,000 µL pipette tip (Thermo Fisher Scientific) which was cut approximately 20 mm from the base and firmly attached to the Push gauge.

**Figure 2:**
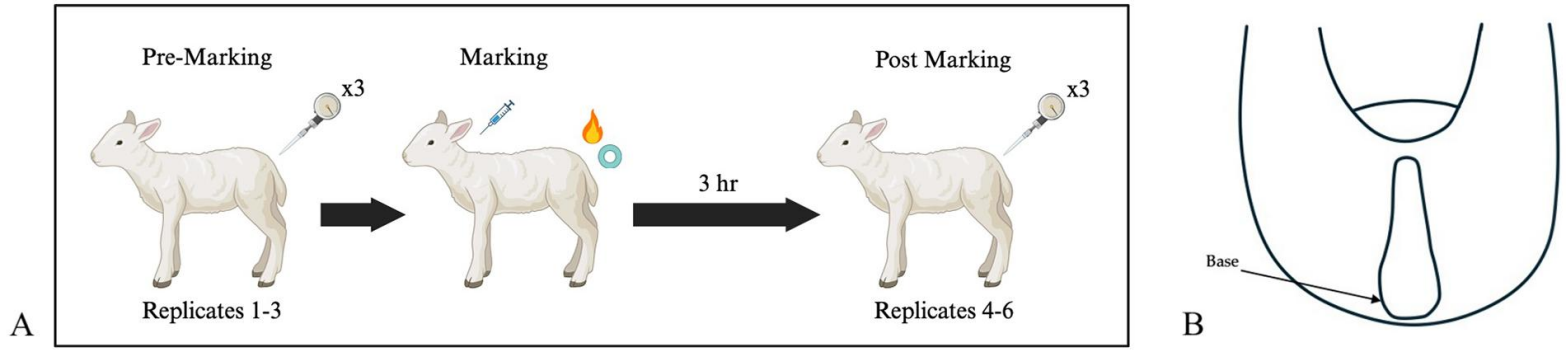
Experimental timeline and test site for mechanical nociceptive threshold (MNT) assessment. Prior to marking, lambs underwent three baseline MNT tests using a modified algometer (replicates 1-3). Lambs were then randomly allocated to a marking group; all lambs were vaccinated and marked according to their assigned group. After marking, lambs were returned to a large holding yard with their mothers for three hours, after which another three MNT tests were performed (replicates 4-6) **(A)** Diagram showing the test site for MNT assessment at the base of the tail **(B)** Adapted from (31). Created in BioRender. Hutchinson, M. (2026) https://BioRender.com/gexvy16.

The study employed a handheld algometer (Wagner Force DialTM FDK Series Push Pull Force Gage, FDK 10; Wagner Instruments, CT, USA, Maximum force 5 kgf) with a modified 1,000 µL pipette tip (Thermo Fisher Scientific) which was cut approximately 20 mm from the base and firmly attached to the Push gauge, referred to as the algometer in this article (Figure 1). The pipette tip was hollow and had an outer diameter of 1.1 mm and an inner diameter of 0.8 mm, with a total area of approximately 0.45 mm^2^. It was regularly inspected throughout the experiment to ensure it was not damaged and that it was changed as needed to maintain integrity. To perform the MNT testing, each lamb was picked up by a handler who used one arm to support the lamb's body in an upright position with the dorsum against the front of the handler, the lamb's rump was stabilized by the handler's other hand and the top fence rail, allowing measurements to be taken at the base of the tail. The operator (SM) would then perform the MNT measurements. SM approached the lamb from the caudal end, and the algometer was applied gradually (1 kgf/s) to the base of the tail to stimulate the tail dermatome (Figure 2). When a positive reaction (twitch, vocalization, or hind leg movement) was observed by the operator the algometer was removed. The force applied was then recorded and the procedure repeated another two times immediately. If there was no response at a maximum pressure of 5kgf, the maximum value of 5.00kgf was recorded. The 5 kgf cutoff was selected to avoid the risk of tissue damage at the testing site. We also aimed to use a robust, commercially available instrument with sufficient measurement range to detect changes in nociceptive threshold in young lambs both before and after a noxious procedure. Based on preliminary testing, an instrument with a cutoff of 5 kgf provided an appropriate range for lambs from three to seven weeks of age without causing tissue damage. The average of these triplicate stimulations was considered a single test (hereafter referred to as a replicate), reflecting the intended method application. For each lamb, this MNT procedure was conducted a total of three times (with at least 1 min, and a maximum of 12 min, between tests) prior to marking, and three times post-marking, for a total of 6 replicates (18 stimulations) (Supplementary Figure S.1). The same experienced investigator (SM) performed the measurement every time. The design of our study required at least three handlers actively selecting and restraining lambs for SM, to avoid excessive strain on the handlers and ensure efficient testing.

The operator SM and handlers were blinded to intervention groups pre-marking. Blinding was limited post-marking as the presence or absence of a tail was visually apparent. SM conveyed raw MNT measurements, as indicated on the algometer, to the scribe, who recorded all measurements on paper.

### 2.5 Statistical analysis

Statistical analysis was performed using R Statistical Software [v4.4.2; (38)] in the RStudio integrated development environment [v2024.9.1.394; (39)]. A *p*-value of less than 0.05 was considered statistically significant for all analyses.

#### 2.5.1 Precision

The within-animal coefficient of variation (CV) and standard deviation (SD) were calculated for each lamb for the three replicates prior to marking, and the three replicates post marking, to assess relative and absolute imprecision, respectively. To assess the effects of marking status (pre-/post-marking), marking group (control, ring docked, knife docked, ring docked + castrated, or knife docked + castrated), sex and weight on relative imprecision, a linear mixed effects model was performed using the *lmer* function of the *lme4* package (40). Marking status by marking group interaction, weight and sex were fixed effects, and animal was a random effect. Natural log (ln) transformation of CV was required to meet model assumptions based on *simulateResiduals* function of the *DHARMa* package (41). Overall significance of fixed effects was assessed by *F*-test with Kenward-Roger approximation for degrees of freedom using the *KRmodcomp* function of *pbkrtest* package (42). If the marking status by group interaction was not significant, significant main effects of marking status and marking group were tested (*F*-test as above) without the interaction term.

Whilst the intended method application employs means of triplicate stimulations per test (replicate), within-animal within-replicate triplicate CVs were also calculated to enable direct comparison with previous studies reporting only CVs of consecutive stimulations (without averaging) on a single occasion.

The impact of different variables on residual variance (absolute precision) was examined using generalized linear mixed effects modelling with the package *glmmTMB* (43). MNT was the dependent variable with a conditional model consisting of marking status by group interaction as a fixed effect and animal as a random effect, and a dispersion model initially consisting of marking status by group interaction, sex, (centered) weight, and (centered) age. Significant associations of variables in the dispersion model with residual variance were determined individually by likelihood ratio test comparison of nested models (with and without a specific dispersion model term) using the *anova* function in the *stats* package (38). If the marking status by marking group interaction term in the dispersion model was not significant, significant effects of marking status and marking group in the dispersion model were tested without the interaction term.

Bland-Altman analysis (44) was used to assess agreement between pairs of replicates, with differences between the replicates for each lamb plotted against the mean of those replicates for each lamb, and limits of agreement calculated as the 95% confidence limits of the overall mean.

A linear mixed effects model was used to test for any systematic effects of replicate or handler on variability in MNT. The model included random effect for animal and fixed effects of marking status by group interaction, replicate, and handler. Overall significance of replicate and handler fixed effects was assessed by *F*-test with Kenward-Roger approximation for degrees of freedom. Relative contributions of handler and replicate to MNT variance were calculated using random effect variance estimates from linear mixed effects regression of MNT with random effects for replicate (random intercept), handler (random intercept) and id (random intercept and random slope for marking status).

#### 2.5.2 Reliability

Pairwise correlations in MNT between the three replicates pre-marking, and three replicates post-marking, were calculated using Spearman's correlation [*cor.test* function of the *stats* package (38)] as the post marking MNT results were not normally distributed.

Intra-operator reliability, accounting for repeated measurements of animals before and after marking, was assessed using linear mixed effects regression of MNT as dependent variable, measurement replicate (1, 2 or 3, as factor) as fixed effect, random intercept for each animal, and random slope for marking effect (pre- vs. post-marking) within each animal. Intra-operator reliability ICC was then calculated as total subject variance (intercept, slope and covariance)/[(total subject variance) + residual variance], with 95% confidence intervals determined by bootstrapping (1,000 replicates) (45, 46).

#### 2.5.3 Minimum detectable change

The standard error of measurement was also calculated using the residual variance from the linear mixed-effects model used to calculate intra-operator reliability. The standard error of measurement was used to calculate the minimum detectable change (MDC) (47).

#### 2.5.4 Response to marking

Linear mixed effects regression was used to test the effect of marking on MNT, utilizing only the first (first pre-marking) and fourth (first post-marking) replicates to reflect the intended field application of the MNT method. A marking status by marking group interaction was the fixed effect, and animal was a random effect. Overall significance of fixed effects was assessed by *F*-test with Kenward-Roger approximation for degrees of freedom and relevant *post-hoc* comparisons were performed using the *emmeans* package (48) with Bonferroni adjustment. If the marking status by group interaction was not significant, significant main effects of marking status and marking group were tested (*F*-test as above) without the interaction term. Although it does not reflect the likely method context of use, for completeness, the same marking effect analysis was also conducted using data from all replicates.

## 3 Results

Forty-six lambs (males = 29, females = 17) were originally recruited into the study. After the third replicate, one lamb was mistakenly returned to the larger flock, and another lamb escaped into the larger flock unassisted. Therefore, forty-four lambs were included in the final analysis; 6 males and 7 females tail docked with hot knife, 6 males and 7 females tail docked with rubber ring, 5 males tail docked with hot knife and castrated with rubber ring, 7 males tail docked and castrated with rubber ring, and 3 males and 3 females sham handled controls.

### 3.1 Measurement range

The median of all replicates of MNT in lambs was 3.07 kgf (IQR = 2–3.84 kgf, range 1.23–5 kgf). Of a total of 792 individual MNT stimulations (including triplicate stimulations for calculation of replicate means), 55 stimulations (44 pre-marking and 11 post marking), across 17 lambs, reached the maximum of 5 kgf without a response (7%). Four of 264 replicate means (1.5%) were at the upper limit of 5 kgf and these were all pre-marking (The distribution of MNT results from all lambs at each replicate is depicted in Supplementary Figure S.1).

### 3.2 Precision

#### 3.2.1 Relative precision (CV)

The median within-animal CV for the three replicates pre- and post-marking across all lambs (*n* = 88 CVs) was 12.50% (IQR = 7.29–18.16%, range = 1.49–55.40%) (Table 1). There was no significant marking status by marking group interaction for ln(CV) [F_(4, 39)_ = 0.53, *p* = 0.71]. There was a significant effect of marking status on ln(CV). CVs were significantly [F_(1, 43)_ = 15.50, *p* = 0.0003] lower prior to marking [estimated marginal mean ± SE ln(CV) = 2.13 ± 0.11] compared to post-marking (2.71 ± 0.11), but not related to sex [Females = 2.33 ± 0.15, Males = 2.52 ± 0.10; F_(1, 37)_ = 1.17, *p* = 0.29], weight [F_(1, 37)_ = 1.84, *p* = 0.18] nor marking group [Control = 2.42 ± 0.20, Knife = 2.38 ± 0.14, Ring = 2.30 ± 0.14, Knife + Castrate = 2.60 ± 0.24, Ring + Castrate = 2.42 ± 0.21; F_(4, 37)_ = 0.32, *p* = 0.86] (Supplementary Figure S.2).

**Table 1:** Median within-animal/marking status relative precision of mechanical nociceptive thresholds (MNT) at the tail base in lambs separated by marking group.

Group	Marking	Median CV (IQR) (%)	Range (%)
Control	Pre	12.74 (9.23)	2.49–22.03
	Post	15.17 (6.22)	5.63–37.84
Knife	Pre	9.43 (6.12)	2.97–22.54
	Post	14.76 (14.23)	3.63–39.40
Ring	Pre	7.93 (7.98)	1.49–21.84
	Post	11.52 (10.18)	3.87–55.40
Knife + Castrate	Pre	12.46 (2.67)	6.07–17.33
	Post	17.46 (9.38)	10.00–43.35
Ring + Castrate	Pre	8.60 (7.60)	1.57–15.38
	Post	19.90 (7.86)	16.92–41.99

CV, Within-animal coefficient of variation of triplicate stimulations performed on each lamb at each replicate; IQR, interquartile range.

The median within-animal CV within each replicate ranged from 8.90% (IQR = 7.15–15.06%) in replicate 2, to 15.29% (IQR = 9.12–20.46%) in replicate 6 (Supplementary Table S.1).

#### 3.2.2 Absolute precision (SD)

Initial analyses indicated greater body weight (dispersion coefficient estimate ± SE = 0.07 ± 0.02; likelihood ratio test, *χ*^2^_(1)_ = 15.5, *p* = 8 × 10^−5^) and male sex (dispersion coefficient estimate ± SE = 0.29 ± 0.13; likelihood ratio test, *χ*^2^_(1)_ = 5.4, *p* = 0.02) were associated with significantly larger residual variance. However, the association between sex and residual variance was entirely dependent on 2 outlying high variance animals (one male ring docked lamb and one male ring docked and castrated lamb with post-marking SD 4.4 and 3.8 standard deviations from the mean SD, respectively; see Supplementary Figure S.3.1). Excluding these outlier values, greater body weight (dispersion coefficient estimate ± SE = 0.07 ± 0.02; likelihood ratio test, *χ*^2^_(1)_ = 15.9, *p* = 7 × 10^−5^), but not sex (dispersion coefficient estimate ± SE = 0.23 ± 0.13; likelihood ratio test, *χ*^2^_(1)_ = 3.3, *p* = 0.07), remained associated with significantly larger residual variance (Supplementary Figure S.3.2 and Supplementary Tables S.2.1, S.2.2).

#### 3.2.3 Agreement

**Figure 3:**
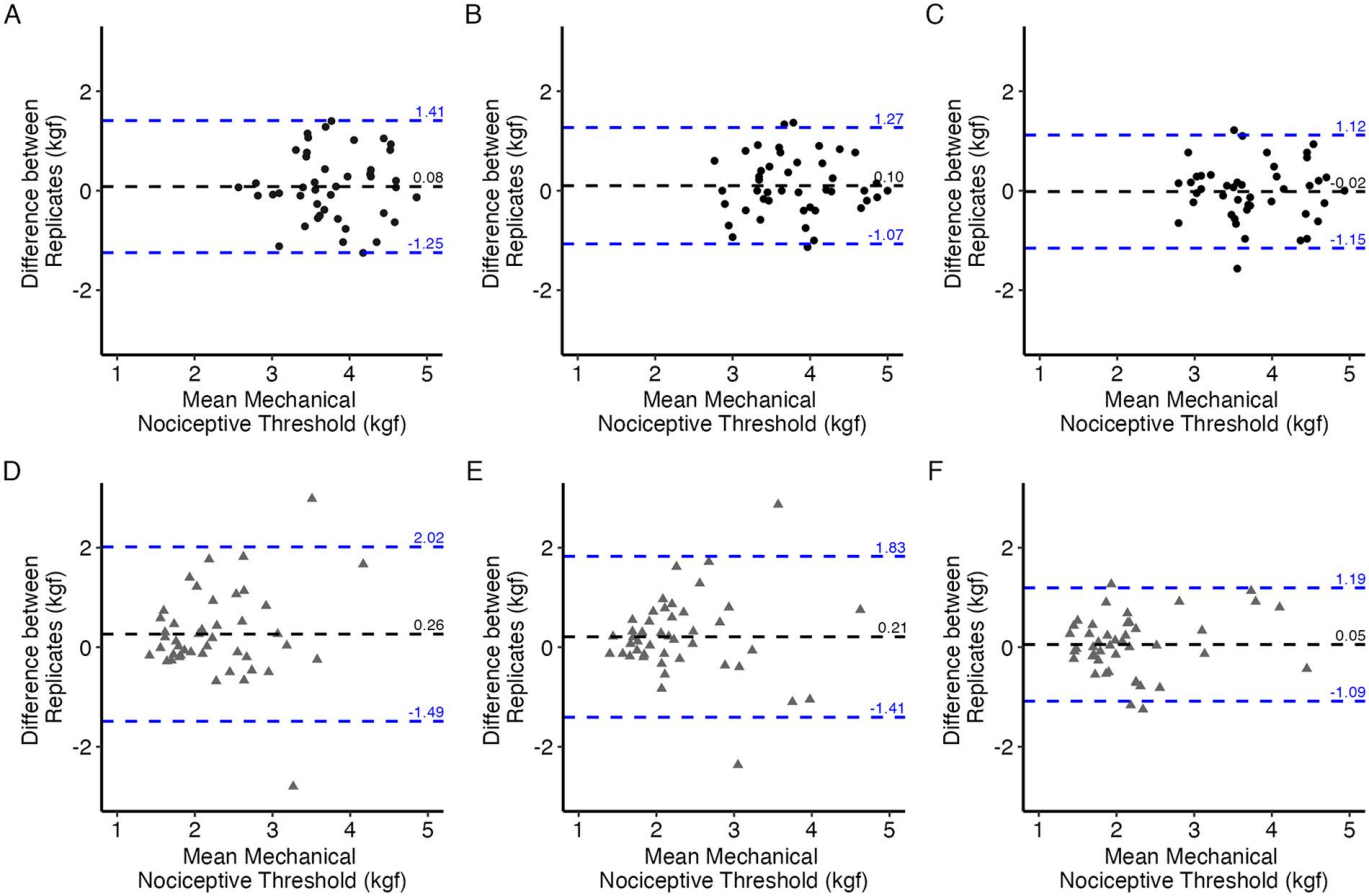
Bland-Altman plots of intra-operator agreement between replicates 1 and 2 (pre-marking •) **(A)**, between 1 and 3 (pre-marking •) **(B)**, and between 2 and 3 (pre-marking •) **(C)**, between 4 and 5 (post-marking ▴) **(D)**, between 4 and 6 (post-marking ▴) **(E)**, and between 5 and 6 (post-marking ▴) **(F)** the difference between tests (mean MNT per lamb obtained in test one compared to test two is plotted on the *y*-axis against the mean [(test 1 + test 2)/2] on the *x*-axis. The overall mean difference between tests is depicted by the central dashed line. The upper and lower limits of agreement are represented by the dashed blue lines.

Bland-Altman plots of differences and limits of agreement between the first, second, and third replicates (replicates 1, 2, and 3 pre-marking and replicates 4, 5, and 6 post-marking) are shown in Figure 3. The mean inter-replicate difference was small for all replicates before marking; 0.08 kgf between replicates 1 and 2, 0.10 kgf between replicates 1 and 3, and −0.02 kgf between replicates 2 and 3. The mean inter-replicate difference after marking was 0.26 kgf between replicates 4 and 5, 0.21 kgf between the replicates 4 and 6, and 0.05 kgf between replicates 5 and 6. The 95% limits of agreement were 2.66 kgf for replicates 1 and 2, 2.34 kgf for replicates 1 and 3, 2.27 kgf replicates 2 and 3, 3.51 kgf for replicates 4 and 5, 3.24 kgf for replicates 4 and 6, and 2.28 kgf for replicates 5 and 6. Replicate differences appeared scale-independent (Figure 3).

There was no significant influence of replicate [F_(1, 210)_ = 2.80, *p* = 0.06] nor handler [F_(3, 228)_ = 1.38, *p* = 0.25] on MNT, with handler and replicate explaining only 0.99% and 0.33% of total variance in MNT, respectively.

### 3.3 Intra-operator reliability

**Figure 4:**
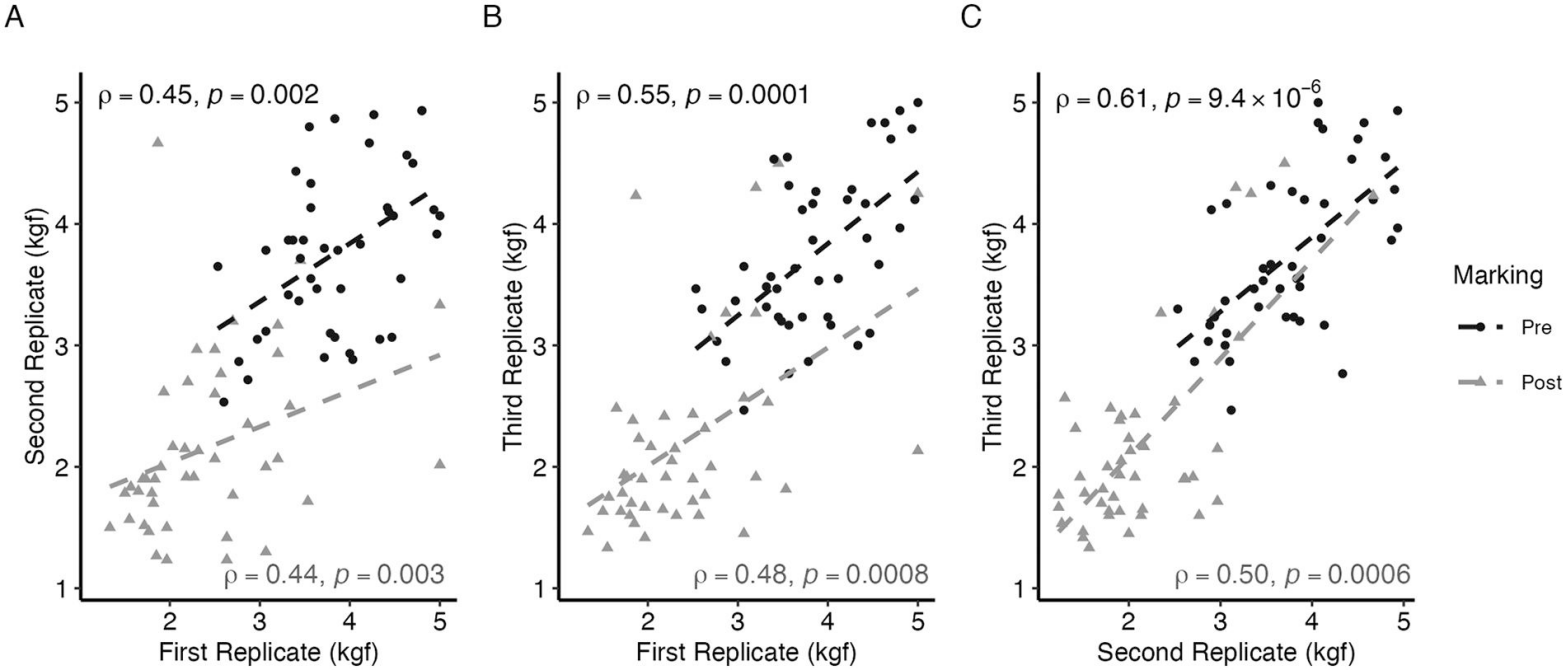
Correlation plots with best-fit linear regression lines (dashed) between MNT from replicates 1 and 2 (pre-marking; •) and between 4 and 5 (post-marking; ▴) **(A)**, between 1 and 3 (pre-marking •) and between 4 and 6 (post-marking ▴) **(B)**, and between 2 and 3 (pre-marking •) and between 5 and 6 (post-marking ▴) **(C)**.

The correlation (Spearman rho) between the first and second replicates was 0.45 (*p* = 0.002) prior to marking and 0.44 (*p* = 0.003) after marking, between the and first and third replicates was 0.55 (*p* = 0.0001) prior to marking and 0.48 (*p* = 0.0008) after marking, and between the second and third replicates was 0.61 (*p* = 9.4 × 10^−6^) before marking and 0.50 (*p* = 0.0006) after marking (Figure 4).

Intra-operator reliability ICC was 0.83 with a 95% confidence interval of 0.81–0.94.

### 3.4 Minimum detectable change and responsivity

**Figure 5:**
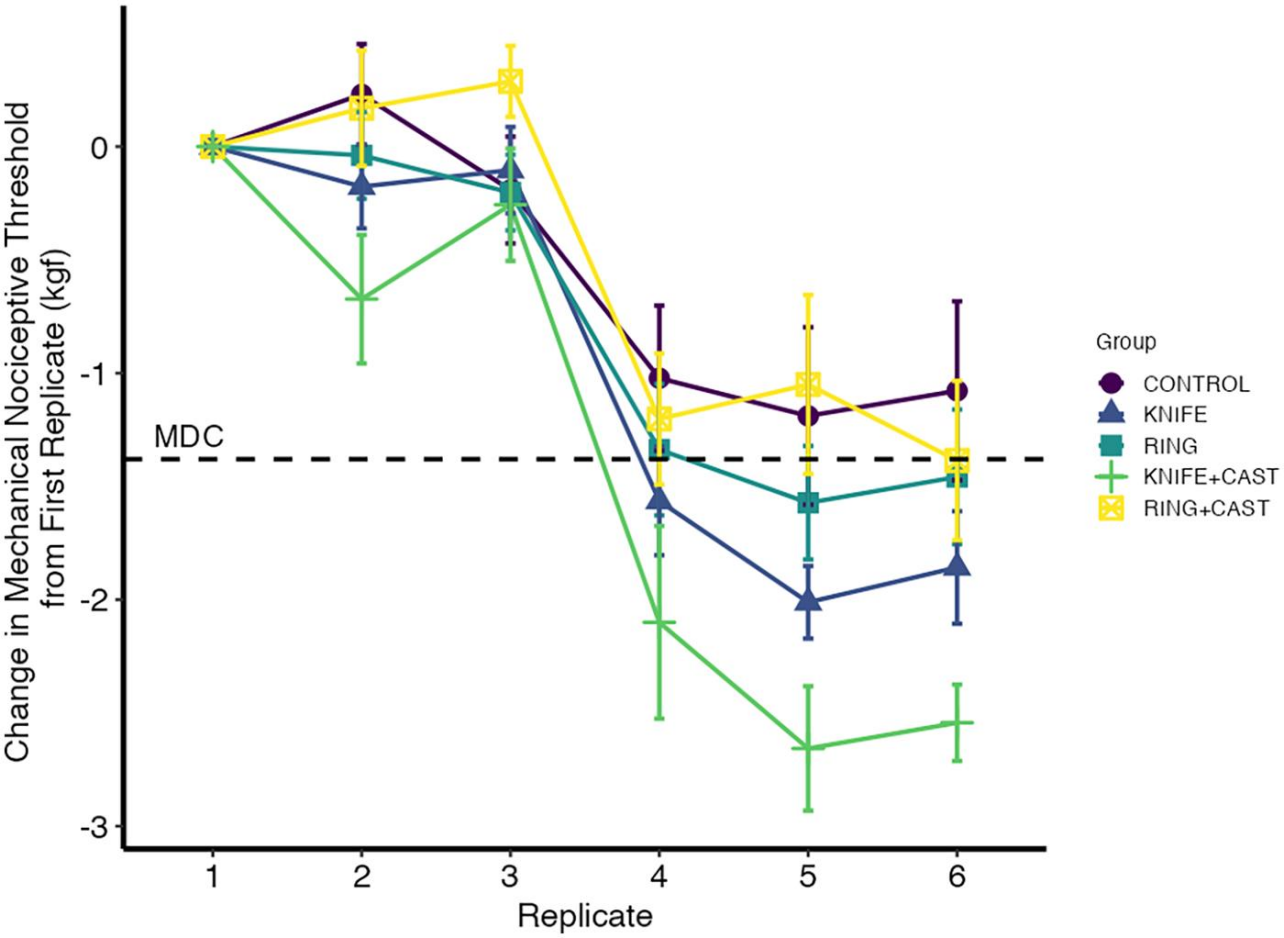
Change in the mean MNT (± sem) for each group at each replicate from the first replicate. Black dashed y-intercept indicates the minimum detectable change (MDC) (1.38 kgf).

The standard error of measurement was 0.50 kgf, with a minimum detectable change of 1.38 kgf (Figure 5).

Using replicate 1 and 4 data only, there was no significant marking status by marking group interaction [F_(4,39)_ = 1.19, *p* = 0.3; Supplementary Table S.3.1]. With the non-significant interaction term removed, there was no significant marking group main effect [F_(4, 39)_ = 1.86, *p* = 0.14]. However, there was a significant main effect of marking (F_(1, 43)_ = 106.11, *p* = 3.48 × 10^−13^; Table 2), with lower MNT post-marking (estimated marginal mean ± SE = 2.5 ± 0.11) vs. pre-marking MNT (3.9 ± 0.11; Figure 5).

**Table 2:** Linear mixed effects regression testing the effect of marking on MNT, utilizing only the first (first pre-marking) and fourth (first post-marking) replicates to reflect the intended field application of the MNT method.

Fixed effects		Estimate (β)	SE	95% CI^b^
(Intercept)^a^		4.19	0.24	3.74 – 4.64
Marking status (post)		−1.43	0.14	−1.70 – −1.15
Marking group	Knife	−0.50	0.23	−1.02 – 0.02
	Knife + Castrate	−0.03	0.34	−0.67 – 0.61
	Ring	−0.55	0.23	−1.08 – −0.03
	Ring + Castrate	−0.15	0.31	−0.74 – 0.44

Random effects	Variance	SD
Animal (Intercept)	0.10	0.32
Residual	0.42	0.65

Model fit	Marginal	Conditional
R^2^	0.52	0.62

*F*-test				
Term	F-statistic	df1	df2	*p*-value
Marking status	106.1	1	43	3 × 10^−13^
Marking group	1.9	4	39	0.1

Model equation: Tail MNT∼Marking status (Replicate 1 or 4)+Marking group + (1|Animal).

SE, Standard Error; SD, standard deviation; df1, numerator degrees of freedom; df2, denominator degrees of freedom.

a Pre-marking control group.

b Profile likelihood confidence intervals.

With inclusion of all replicate data pre- and post-marking, there was a significant marking status by group interaction [F_(4,215)_ = 4.54, *p* = 0.002; Supplementary Table S.3.2], with a greater effect of marking in knife docked and castrated lambs [estimated marginal mean ± SE change (post-pre) in MNT = −2.12 ± 0.20] compared to controls [−1.11 ± 0.19; *post-hoc t*_(215)_ = 3.67, *p* < 0.01; Supplementary Table S.3.3a]. However, all marking groups showed significant (post-hoc *p* < 0.0001) reduction in MNT post-marking (see Supplementary Table S.3.3b). Comparing the difference in MNT between groups either before or after marking showed no difference between any docked groups vs. controls pre-marking (post-hoc *p* = 1). After marking, knife docked lambs had a significantly lower MNT (estimate marginal mean MNT ± SE = 1.9 ± 0.1) compared to controls [2.9 ± 0.2; *post-hoc t*_(62)_ = −3.84, *p* < 0.01; Supplementary Table S.3.3c].

## 4 Discussion

This study tested the feasibility and reliability of using a modified algometer to measure mechanical nociceptive thresholds at the base of the tail in sheep in a field environment using a single experienced operator. We investigated levels of measurement precision, intra-operator reliability and agreement, as well as responsiveness to husbandry procedures. The test demonstrated good reliability, with a minimum detectable change/difference sufficient for the intended context of use in field assessment of the impact of marking procedures on nociceptive thresholds.

### 4.1 Precision, agreement, and reliability

Median within-animal/-occasion CV in this study was 12.5% but ranged widely (1.5–55.4%). Other studies investigating the precision of mechanical nociceptive testing in livestock species have reported higher mean CVs, but were based on repeat stimulations on a single occasion. For example, Raundal, et al. (49) reported a mean within-animal CV of 34% and 41% for five algometer MNT measurements by two operators (one experienced, one inexperienced) at dorsal and lateral sites, respectively, on the cannon bone in dairy cattle. In adult sows, mean CVs of five repeated MNT measurements, performed by the same operator, on the limb was 28.1% (across thoracic and pelvic limbs and using either an actuator or a hand-held probe) and 38.9% on the tail using a hand-held probe (16). In piglets, three MNT measurements at three different tail sites and at 4 different ages, performed by a single operator, resulted in mean within-animal CVs between 30.1% and 32.6% (10). To enable direct comparison, we also calculated within-animal within-replicate triplicate stimulation CV, observing mean CVs from 10.4%–16.8% across replicates, but a total range of 0.0–60.5% (Supplementary Table S.1).

The use of repeated measurements to establish a mean MNT is commonly adopted in quantitative sensory testing methods (30, 32, 49–53), although not always (22, 54). Taking an average of three MNT measurements has been shown to improve reliability over a single measurement for algometer MNT assessment over the thorax in dairy heifers (55) and was therefore chosen as the measurement approach in the current study.

In terms of potential contributors to measurement imprecision, body weight was the only variable that significantly and robustly influenced absolute precision (Supplementary Figures S.3.1, S.3.2). The cause for higher absolute imprecision in heavier lambs is unknown but could be associated with increased difficulty in lifting and securing heavier, and potentially stronger lambs that are less compliant with handling. This could have implications for applying the technique over longer time frames, and in older sheep, where weight gain may influence measurement precision and therefore MDC.

Whilst CV prior to marking was significantly lower than CV post marking, this may be due to the common observation of lower CVs at higher mean values (with higher MNT pre-marking, see Supplementary Figure S.2B) rather than a true effect of the marking procedure. The absence of a significant difference in absolute precision between pre- and post-marking also supports this interpretation, rather than an effect of the stress and pain of marking *per se* on MNT measurement precision.

Bland-Altman plots indicated minimal bias between replicates within pre- and post-marking assessments, supported by the absence of significant replicate effects in regression analyses. The limits of agreement ranged from −1.49 to 2.02 kgf, indicating considerable variability between replicates.

In terms of reliability, there were significant positive correlations between replicates (Spearman's rho 0.44–0.61), and the method showed very good intra-operator reliability (ICC = 0.83), indicating good reliability for repeatable ranking of test subjects. In comparison, MNT at different tail sites in piglets of different ages previously demonstrated only poor to fair intra-operator, within-session reliability (ICC = 0.10–0.59 depending on tail site and age) (10). Whether these differences in reliability relate to differences in species, testing site, device or other factors is unclear. Stubsjøen, et al. (56) assessed the use of an algometer to measure MNT over multiple days on different forelimb locations in 2- to 4-year-old ewes but did not report reliability or agreement. Inclusion of multiple anatomical sites incorporates additional variables, such as the order of testing each site and the anatomical location, be it over a boney prominence or muscular region. Both of which have been found to influence MNT measurement (57, 58). In this study, a single anatomical site was used for testing MNT, so this may have contributed to the improved reliability reported in comparison to other studies. To the best of our knowledge there have been no prior agreement nor reliability studies of MNT testing in lambs with which we can compare.

### 4.2 Minimal detectable change and responsivity to marking

While not the primary focus of this study, the change in MNT following marking was included in the analysis to reflect the intended application of this technique, and to indicate responsiveness and provide context to the MDC. Replicates 1 and 4 were selected to assess marking effects as they represent the approach that would be used in trials of assessing MNT before and after marking. MNT significantly decreased post-marking (*p* < 0.0001) (Figure 5). Notably, only the effects of knife docking (with or without castration) exceeded the MDC of 1.38 kgf when comparing replicates 1 and 4. This does not necessarily mean significant changes in other groups do not reflect true underlying marking effects on nociception, but that the method may have limited precision for reliably detecting these smaller MNT changes within individual lambs. Increasing the number of test replicates, especially post-marking, may further enhance the ability to detect changes in MNT (Figure 5). When all replicates were included in the analysis, a significant marking status by marking group interaction was identified, with knife docked and castrated lambs having a significantly larger reduction in MNT after marking compared to controls (Supplementary Table S.3.3a), in addition to the significant reduction in MNT after marking within all groups (Supplementary Table S.3.3b) as was seen when just Replicates 1 and 4 were used. However, in practice, performing these repeat nociceptive threshold tests at each study time point (required here for assessment of precision, agreement and reliability) is rarely feasible in the field and in large-scale studies.

A reduction in MNT at the base of the tail following tail docking procedures is expected due to induction of secondary hyperalgesia. Similar outcomes have been reported by Lomax, et al. (28), who identified hyperalgesia at skin sites surrounding the tail wound of lambs that were hot knife tail docked and surgically castrated at 6- to 12-weeks of age. Two studies assessed wound sensitivity at the base of the tail in 6- to 10-week-old female lambs 4, 7, and 10 days after mulesing and hot knife tail docking, reporting a significant reduction in nociceptive threshold compared to sham handled controls across all timepoints (31, 59). While differences in the methods of sensory assessment, castration technique, inclusion of mulesing, location of testing, and restraint limit direct comparison, the findings point to comparable changes in nociceptive sensitivity in the first few hours (28) to days (31, 59) after tail docking. In contrast, in 3- to 4-day old lambs, Clark, et al. (32) reported increased MNT (hypoalgesia) at the base of the tail from one to 24 h following ring castration and ring tail docking. These differing outcomes may reflect variation in technique (von Frey vs. algometer), or age differences, with electroencephalogram studies demonstrating developmental increases in cerebro-cortical response to ring castration during the first 10 days of life (60, 61).

In this study, lambs ranged in age from 23 to 52 days. Whilst developmental differences in nociceptive processing may influence responses to noxious stimuli across early life stages (14, 21, 62, 63), previous studies in lambs have reported minimal differences in behavioral and physiological responses to marking procedures across comparable age ranges (64, 65). More importantly, the age range of the lambs used in this study reflects typical Australian production systems [where rams are joined with ewes over approximately 5 weeks and all lambs can be marked as a cohort when the youngest lambs reach an appropriate age (66)]. Therefore, the age range reflects the context of use and is a strength of the study in providing a realistic assessment of the MNT method performance under conditions relevant to its intended application.

The current study also observed significantly reduced MNT following sham marking in control lambs. Given the absence of significant replicate differences within pre- and post-marking occasions, and the break of at least three hours between pre- and post-marking, it seems unlikely that this change in control lambs is a training or habituation effect (16, 49). Alternatively, it is possible that the reduction in MNT from pre- to post-marking in controls could be related to other factors distinct from tissue injury-related hyperalgesia. These factors may include heightened nociceptive sensitivity associated with vaccination (67–69), and non-specific stressors such as handling, restraint, and temporary separation from the dam (32, 70). Reduced MNT following vaccination may be related to the development of myalgia due to increased expression of inflammatory mediators (71). Stress associated with other non-marking study factors (e.g., handling, separation and other environmental factors discussed above) has been reported to both increase (72) or decrease nociceptive thresholds (32, 73). For example, Clark, et al. (32) found that very young lambs separated from their mother for 10 min developed hyperalgesia three hours after the stressful event. Whatever the underlying cause, this highlights the necessity of appropriate control groups for investigating marking procedure effects on nociception.

The primary aim of this study was to assess the precision, reliability, and agreement of the method within the context of lamb marking. Now that the method has been demonstrated to perform reliably, future larger studies with appropriate power can be performed to explore the influence of marking procedures and additional variables, including vaccination, age, sex, weight, and analgesic interventions, on MNT in lambs.

### 4.3 Context of use and feasibility

#### 4.3.1 Environment, handling, restraint and operator

The intended application of the MNT method being evaluated in this study involves measurement in sheep yards at the time of marking, which is typically a busy and noisy period. The sheep yards can be a stressful environment, particularly for lambs that have not been exposed to that space before and may never have been moved away from their home paddock or physically separated from their mother. Noise, particularly unfamiliar or loud noise, can be stressful to livestock and create distraction (74). Even white noise has been shown to impede cognitive performance in sheep navigating a spatial maze (75). Background noise is unavoidable in the sheep yards, especially during marking. A combination of human communication and movement within the yards, and lambs and ewes vocalizing due to separation or pain, can substantially increase noise levels and may introduce further distraction during testing. In addition, the method of MNT testing employed in this study requires restraint of the subject, which is known to induce stress (76) and may alter nociceptive sensitivity (72). The restraint method required the lambs to be held in an upright position, which is not a typical position for a lamb. Other studies using quantitative sensory testing have avoided lifting lambs for the procedure to reduce stress by allowing them to stand while restrained (30). In preliminary tests, we found it more difficult to observe subtle hind leg movements in response to sensory testing while the subject was standing. Holding the lambs, rather than having them standing or restrained in a marking cradle (29, 31, 77), allowed free movement of the hind legs, making responses to the algometer application easier to detect. In terms of feasibility, all lambs (weighing up to 18.6 kg) were able to be lifted and adequately restrained for testing in this study. However, this method of restraint would be difficult in heavier lambs.

Different handlers may also contribute to imprecision in MNT testing due to factors such as experience with sheep, strength, odorants of clothes, sex, and pitch and volume of voice. Multiple handlers were necessary in this study to increase efficiency, minimize the time lambs were separated from their mothers, and to mitigate manual handling risks. Therefore, any variability or bias introduced by multiple handlers is accepted as part of the measurement imprecision, noting also that handler had no systematic impact (bias) on MNT in this study, explaining less than 1% of total variance in MNT measurements.

This study used a single operator, which matched our intended context of use for this method in our broader research. Whilst an entirely objective assessment of MNT is desired, it is not possible within the practical limitations of the method, instrument, and environment. For example, to monitor if maximum pressure (5 kgf) had been reached, the operator could not be completely blinded to the algometer reading whilst assessing animal reactions. In addition, necessary identification markings on lambs and communication between handlers and scribe can unblind the operator to the identity of each animal, which is relevant for repeat testing. The operator was also not blinded to marking group post-marking, due to clear presence or absence of elastrator rings and/or intact tails. Finally, the method ultimately requires the operator's assessment of whether a reaction has been elicited from the animal, thus also affecting measurement objectivity.

Therefore, while not providing ideal conditions for objective nociceptive threshold testing, all of the above environmental, handling, restraint and operator conditions were necessary to enable a valid assessment of the MNT method performance under conditions relevant to its intended application.

#### 4.3.2 Instrumentation

The algometer and pipette combination presents a relatively cheap and widely available instrument setup that can be used in a range of environments and is less susceptible to damage through rough handling or interaction with animals compared to more traditional von Frey filaments. The algometer and pipette tip system was robust enough to withstand use in the setting of sheep yards. The pipette tips bent in some cases after application of maximum force (5 kgf), this was closely monitored and the tip changed regularly to ensure a consistent tip diameter and force was applied. The use of a P1000 pipette tip provides a small contact area, focusing mechanical pressure on a confined point and rapidly increasing pressure with relatively small increases in force. Smaller probes elicit more skin stress and typically assess superficial somatic pain when used to measure nociceptive thresholds. Large probes, require greater force to apply the same pressure and activate nociceptors from deeper tissues including adipose and muscle assessing deep tissue sensitivity (54, 78). Probes with a larger surface area have been shown to result in higher thresholds (force applied) and reduced data consistency (79).

The rate of application of force may contribute to variability in the results as faster rates have been shown to lead to significantly higher MNT measurements in the same animals (17, 33, 79). In this study, the rate of application was controlled by the operator using observation of the algometer dial where possible and based on experience. Rate of application of force may have acted as a confounding factor, although the use of a single experienced operator was adopted to limit its influence. The use of specialized algometers which provide feedback on rate of application to ensure consistency would reduce the influence of application rate on measurements (16, 17, 33, 57). However, these devices are more costly and delicate compared to the algometer used in the study, which could limit their utility in a marking environment. In future studies to refine the method, particularly studies on inter-rater reliability, using devices that ensure an accurate and consistent rate of application of force across operators would be beneficial.

### 4.4 Limitations

This study was designed to assess the precision, reliability, and agreement of the MNT method in a real-world setting (lamb marking). As such, contextual factors such as background noise, and handling, were inherent to the method being assessed. However, some limitations were introduced which may influence the findings.

One constraint was the limited time available to obtain the necessary number of test repetitions to achieve appropriate statistical power, while minimizing handling stress, separation from the dam (lamb's mother), and ensuring adequate time for the lambs to recover from the marking procedures prior to returning to their paddocks. A minimum interval of 2 min between replicates within the pre- and post-marking blocks of replicates was set based on subject numbers, and operator efficiency. While the median inter-replicate interval was three minutes, in some instances the inter-replicate interval was less than two minutes due to the random selection of lambs by multiple handlers and operational time pressures. Nalon, et al. (16) found no significant effect of a one- vs. three-minute measurement interval on MNT in healthy sows, although MNT tended to increase across four to five successive measurements, potentially due to habituation (49). In adult sheep, repeated MNT testing at 5 min intervals over 30 min resulted in reduced MNT with successive repetitions, suggesting a learned response or sensitization (56). In the present study, MNT did not differ significantly across three replicates within either the pre- or post-marking blocks. It is possible that differences in subjects (young lambs) and technique (manual restraint for three replicates), mitigated habituation or learned responses observed in other studies. Critically, for the intended use of the method replicates would be separated by approximately three hours, making any potential effects of very short inter-replicate intervals unlikely to be relevant.

Another limitation was that this study assessed only a single experienced operator. Therefore, method performance cannot necessarily be generalized beyond this study to different operators. Additionally, operator experience can have a significant influence on MNT results (57). In future, studies employing multiple operators would also need to assess inter-operator reliability and consider operator experience.

Finally, while the reduction in MNT in sham-handled lambs raises the possibility of an effect of vaccination (and the inherent immune response) on nociceptive sensitivity as discussed in Section 4.2, non-vaccinated controls could not be included in this study due to limitations in subject number and time constraints. Additionally, the practical and ethical feasibility of withholding vaccinations during lamb marking limited the use of such a control group in this setting. However, future studies could include unvaccinated controls to explore the influence of vaccination on MNT, potentially guiding vaccine administration protocols during this time.

## 5 Conclusions

In conclusion, measuring the mechanical nociceptive threshold at the base of the tail in manually restrained lambs proved to be feasible, have good reliability, and be responsive to husbandry procedures expected to induce hyperalgesia, under practical field conditions. The technique presents a practical, low-cost tool to assess mechanical nociceptive consequences of husbandry procedures in young lambs. The availability of reliable, field-adaptable methods for assessing nociceptive thresholds will aid in understanding the short- and long-term nociceptive consequences of painful procedures and testing the efficacy of analgesics.

## Data Availability

The original contributions presented in the study are included in the article/Supplementary Material, further inquiries can be directed to the corresponding authors.
