# Supplementary material for: Feasibility and reliability of pressure algometry for mechanical nociceptive threshold quantification in lambs in a field environment
Source: Front Pain Res (Lausanne). 2026 Jun 24;7:1696631. doi: 10.3389/fpain.2026.1696631 (PMC13341597; doi:10.3389/fpain.2026.1696631)
Supplement: Supplementary file 1 [file Datasheet1.zip › Supplementary materials/Supplementary Tables.docx]

Supplementary Tables

Table S.1 | Within-replicate triplicate stimulation relative precision of mechanical nociceptive thresholds (MNT) at the tail base in lambs.

| Replicate | Median CV (IQR) (%) | Mean CV (± SEM) (%) | Range (%) |
| --- | --- | --- | --- |
| 1 | 8.9 (5.4-14.2) | 10.4 (± 0.6) | 0.0-31.3 |
| 2 | 8.8 (7.1-15.1) | 10.4 (± 0.5) | 2.0-23.4 |
| 3 | 9.8 (6.2-17.7) | 11.6 (± 0.6) | 0.0-26.4 |
| 4 | 13.9 (8.6-19.3) | 14.3 (± 0.7) | 0.0-32.4 |
| 5 | 14.3 (9.5-21.8) | 16.8 (± 1.0) | 1.9-60.5 |
| 6 | 15.3 (9.1-20.5) | 15.6 (± 0.6) | 2.4-31.3 |

CV: Within-animal coefficient of variation of triplicate stimulations performed on each lamb at each replicate.
IQR: interquartile range. SEM: Standard error of the mean.

**Table S.2.1 | Summaries of combine conditional and** dispersion models examining the influence of marking status by marking group interaction, sex, and weight on residual variance in all animals and excluding two outlier animals.

| Conditional model: | | All animals | | | | Excluding outliers^c^ | |
| --- | --- | --- | --- | --- | --- | --- | --- |
|  |  | **Estimate** | | **SE** | | **Estimate** | **SE** |
| (Intercept)^a^ | | 3.96 | | 0.19 | | 3.98 | 0.20 |
| Marking status (post) | | -1.12 | | 0.18 | | -1.12 | 0.18 |
| Marking group | |  | |  | |  |  |
|  | Knife | -0.29 | | 0.23 | | -0.28 | 0.24 |
|  | Knife+Castrate | 0.19 | | 0.28 | | 0.20 | 0.29 |
|  | Ring | -0.47 | | 0.22 | | -0.48 | 0.23 |
|  | Ring+Castrate | 0.09 | | 0.25 | | 0.10 | 0.27 |
| Marking status:marking group | |  | |  | |  |  |
|  | Post:Knife | -0.67 | | 0.21 | | -0.67 | 0.21 |
|  | Post:Knife+Castrate | -1.00 | | 0.27 | | -1.01 | 0.27 |
|  | Post:Ring | -0.37 | | 0.21 | | -0.40 | 0.20 |
|  | Post:Ring+Castrate | -0.39 | | 0.27 | | -0.49 | 0.26 |
| Dispersion model: | |  |  | | |  | |
|  |  | **Estimate** | | | **SE** | **Estimate** | **SE** |
| (Intercept)^b^ | | -0.62 | | | 0.20 | -0.61 | 0.21 |
| Marking status (post) | | -0.03 | | | 0.29 | -0.03 | 0.29 |
| Marking group | |  |  |  |  |  |  |
|  | Knife | -0.11 | | | 0.24 | -0.09 | 0.24 |
|  | Knife+Castrate | -0.27 | | | 0.30 | -0.22 | 0.31 |
|  | Ring | -0.32 | | | 0.24 | -0.21 | 0.25 |
|  | Ring+Castrate | -0.64 | | | 0.28 | -0.54 | 0.29 |
| Sex (male) | | 0.29 | | | 0.13 | 0.23 | 0.13 |
| Weight (centered) | | 0.07 | | | 0.02 | 0.07 | 0.02 |
| Marking status:marking group | |  |  |  |  |  |  |
|  | Post:Knife | -0.31 | | | 0.35 | -0.28 | 0.36 |
|  | Post:Knife+Castrate | 0.01 | | | 0.42 | 0.02 | 0.43 |
|  | Post:Ring | 0.21 | | | 0.36 | -0.16 | 0.38 |
|  | Post:Ring+Castrate | 0.62 | | | 0.38 | 0.42 | 0.40 |
|  |  |  | | |  |  |  |
| Random effects (conditional model) | | **Variance** | | | **SD** | **Variance** | **SD** |
| Animal (Intercept) | | 0.11 | | | 0.33 | 0.12 | 0.35 |

^a^Pre-marking control group. ^b^Pre-marking control females (centered weight = 0). ^c^ Excluding two outlying high variance animals (one male ring docked lamb and one male ring docked and castrated lamb with post-marking SD 4.4 and 3.8 standard deviations from the mean SD, respectively).
Model formulation is conditional model of tail MNT ~ marking status (pre or post marking, including all replicates)*marking group + (1|Animal), dispersion model of ~ marking status * marking group + sex + weight (centered), and gaussian family with identity link. SE: Standard error of co-efficient estimate.

**Table S.2.2** | Likelihood ratio test comparisons of nested models with or without specific dispersion model terms in all animals and excluding outlier animals.

|  | **Dispersion term assessed** | **Full dispersion model** | **Reduced dispersion model** | **Chi-square (df)** | **p-value** |
| --- | --- | --- | --- | --- | --- |
| **All animals** | Marking status-by-marking group interaction | marking status*marking group + sex + weight | sex + weight | 8.0 (4) | 0.09 |
|  | Marking status | marking status + marking group + sex + weight | marking group + sex + weight | 0.3 (1) | 0.6 |
|  | Marking group | marking status + marking group + sex + weight | marking status + sex + weight | 2.6 (4) | 0.6 |
|  | Sex | marking status*marking group + sex + weight | marking status*marking group + weight | 5.4 (1) | 0.02 |
|  | Weight | marking status*marking group + sex + weight | marking status*marking group + sex | 15.5 (1) | 8x10^-5^ |
| **Excluding outliers^a^** | Marking status-by-marking group interaction | marking status*marking group + sex + weight | sex + weight | 4.2 (4) | 0.4 |
|  | Marking status | marking status + marking group + sex + weight | marking group + sex + weight | 0.4 (1) | 0.5 |
|  | Marking group | marking status + marking group + sex + weight | marking status + sex + weight | 3.5 (4) | 0.5 |
|  | Sex | marking status*marking group + sex + weight | marking status*marking group + weight | 3.3 (1) | 0.07 |
|  | Weight | marking status*marking group + sex + weight | marking status*marking group + sex | 15.9 (1) | 7x10^-5^ |

All models have conditional model MNT ~ marking status * marking group + (1|Animal), gaussian family with identity link. ^a^Excluding two outlying high variance animals (one male ring docked lamb and one male ring docked and castrated lamb with post-marking SD 4.4 and 3.8 standard deviations from the mean SD, respectively). df: degrees of freedom.

**Table S.3.1** | Linear mixed effects regression testing the effect of marking on MNT, utilizing only the first (first pre-marking) and fourth (first post-marking) replicates to reflect the intended field application of the MNT method.

| Fixed effects |  | Estimate (β) | SE | 95% CI^b^ |
| --- | --- | --- | --- | --- |
| (Intercept)^a^ |  | 3.99 | 0.30 | 3.44 – 4.54 |
| Marking status (post) | | -1.02 | 0.37 | -1.72 – -0.32 |
| Marking group | |  |  |  |
|  | Knife | -0.23 | 0.36 | -0.90 – 0.44 |
|  | Knife+Castrate | 0.51 | 0.44 | -0.31 – 1.32 |
|  | Ring | -0.40 | 0.36 | -1.06 – 0.27 |
|  | Ring+Castrate | -0.06 | 0.40 | -0.81 – 0.69 |
| Marking status x marking group | |  |  |  |
|  | Post:Knife | -0.54 | 0.45 | -1.39 – 0.31 |
|  | Post:Knife+Castrate | -1.08 | 0.55 | -2.12 – -0.04 |
|  | Post:Ring | -0.32 | 0.45 | -1.16 – 0.53 |
|  | Post:Ring+Castrate | -0.18 | 0.51 | -1.14 – 0.78 |

| Random effects | Variance | SD |
| --- | --- | --- |
| Animal (Intercept) | 0.11 | 0.33 |
| Residual | 0.41 | 0.64 |

| Model fit |  |  |  |
| --- | --- | --- | --- |
| R^2^ | **Marginal** |  | **Conditional** |
|  | 0.53 |  | 0.63 |

| F-test |  |  |  |  |
| --- | --- | --- | --- | --- |
| Term | **F-statistic** | **df1** | **df2** | **p-value** |
| Marking status*marking group | 1.19 | 4 | 39 | 0.3 |

^a^Pre-marking control group. ^b^Profile likelihood confidence intervals.
Model equation: Tail MNT ~ Marking status (Replicate 1 or 4)*Marking group + (1|Animal).
SE: Standard Error. SD: standard deviation. df1: numerator degrees of freedom. df2: denominator degrees of freedom.

**Table S.3.2** | Linear mixed effects model showing the effect of marking status, marking group, and marking status by group interaction on mechanical nociceptive threshold at the base of the tail in lambs using all replicate data.

| Fixed effects |  | Estimate (β) | SE | 95% CI^b^ |
| --- | --- | --- | --- | --- |
| (Intercept)^a^ |  | 4.00 | 0.20 | 3.61 – 4.38 |
| Marking status (post) | | -1.11 | 0.19 | -1.47 – -0.75 |
| Marking group | |  |  |  |
|  | Knife | -0.34 | 0.25 | -0.80 – 0.13 |
|  | Knife+Castrate | 0.18 | 0.30 | -0.39 – 0.76 |
|  | Ring | -0.49 | 0.25 | -0.96 – -0.02 |
|  | Ring+Castrate | 0.08 | 0.28 | -0.45 – 0.60 |
| Marking status x marking group | | |  |  |
|  | Post:Knife | -0.61 | 0.23 | -1.05 – -0.17 |
|  | Post:Knife+Castrate | -1.01 | 0.28 | -1.55 – -0.48 |
|  | Post:Ring | -0.27 | 0.23 | -0.70 – 0.17 |
|  | Post:Ring+Castrate | -0.26 | 0.25 | -0.75 – 0.24 |

| Random effects | Variance | SD |
| --- | --- | --- |
| Animal (Intercept) | 0.14 | 0.38 |
| Residual | 0.31 | 0.56 |

| Model fit |  |  |  |
| --- | --- | --- | --- |
| R^2^ | **Marginal** |  | **Conditional** |
|  | 0.60 |  | 0.73 |

| F-test |  |  |  |  |
| --- | --- | --- | --- | --- |
| Term | **F-statistic** | **df1** | **df2** | **p-value** |
| Marking status*marking group | 4.54 | 4 | 215 | 0.002 |

^a^ Pre-marking control group. ^b^Profile likelihood confidence intervals.
Model equation: Tail MNT ~ Marking status (pre or post marking, including all replicates)*Marking group + (1|Animal).
SE: Standard Error. SD: standard deviation. df1: numerator degrees of freedom. df2: denominator degrees of freedom.

**Table S.3.3a** | Marking status by marking group contrasts from linear mixed effects model comparing the effect of marking procedures on tail base mechanical nociceptive thresholds (MNT) between groups using all replicate data.

| Contrast^a^ | Mean difference (SE) | p value^b^ |
| --- | --- | --- |
| (Post knife - Pre knife) - (Post control - Pre control) | -0.6 (0.2) | 0.07 |
| (Post knife+castrate - Pre knife+castrate) - (Post control - Pre control) | -1.0 (0.3) | 0.003 |
| (Post ring - Pre ring) - (Post control - Pre control) | -0.3 (0.2) | 1 |
| (Post ring+castrate - Pre ring+castrate) - (Post control - Pre Control) | -0.3 (0.3) | 1 |

^a^Derived from model MNT ~ marking status*marking group + (1|Animal). ^b^Bonferroni-adjusted post-hoc p-value. Post: post-marking status. Pre: pre-marking status

**Table S.3.3b** | Marking status by marking group contrasts from linear mixed effects model showing the effect of marking procedures on tail base mechanical nociceptive thresholds (MNT) within marking groups using all replicate data.

| Marking group | Mean difference in MNT from pre-marking to post-marking (SE) | p value^b^ |
| --- | --- | --- |
|  |  |  |
| Control | 1.1 (0.2) | <0.0001 |
| Knife | 1.7 (0.1) | <0.0001 |
| Ring | 1.4 (0.1) | <0.0001 |
| Knife+Castrate | 2.1 (0.2) | <0.0001 |
| Ring+Castrate | 1.4 (0.2) | <0.0001 |

^a^Estimated marginal means from the linear mixed effects regression MNT ~ (marking group)*(pre- versus post-marking, including all replicates) with animal random effect (intercept). ^b^Bonferroni-adjusted post-hoc p-value for pre- versus post-marking within each marking group.
F: female. M: male. MNT: mechanical nociceptive threshold. SE: Standard error.

**Table S.3.3c** | Marking status by marking group contrasts from linear mixed effects model showing the effect of marking group on mechanical nociceptive threshold at the base of the tail before (pre) and after (post) marking using all replicate data.

| **Marking status^a^** | **Group contrast** | **Mean difference (SE)** | **p value^b^** |
| --- | --- | --- | --- |
| Pre | Knife – Control | -0.34 (0.25) | 1 |
| Pre | (Knife+Castrate) – Control | 0.18 (0.30) | 1 |
| Pre | Ring – Control | -0.49 (0.25) | 1 |
| Pre | (Ring+Castrate) – Control | 0.08 (0.28) | 1 |
| Post | Knife – Control | -0.95 (0.25) | 0.007 |
| Post | (Knife+Castrate) – Control | -0.83 (0.30) | 0.2 |
| Post | Ring – Control | -0.76 (0.25) | 0.08 |
| Post | (Ring+Castrate) – Control | -0.18 (0.28) | 1 |

^a^ Marking status (Pre = Replicates 1, 2, and 3; Post = Replicates 4, 5, and 6). ^b^Bonferroni-adjusted post-hoc p-value for difference in MNT between groups pre- and post-marking.
SE: Standard error.
