# Supplementary material for: Feasibility and reliability of pressure algometry for mechanical nociceptive threshold quantification in lambs in a field environment
Source: Front Pain Res (Lausanne). 2026 Jun 24;7:1696631. doi: 10.3389/fpain.2026.1696631 (PMC13341597; doi:10.3389/fpain.2026.1696631)
Supplement: Supplementary file 1 [file Datasheet1.zip › Supplementary materials/Supplementary Figures.docx]

Supplementary Figures


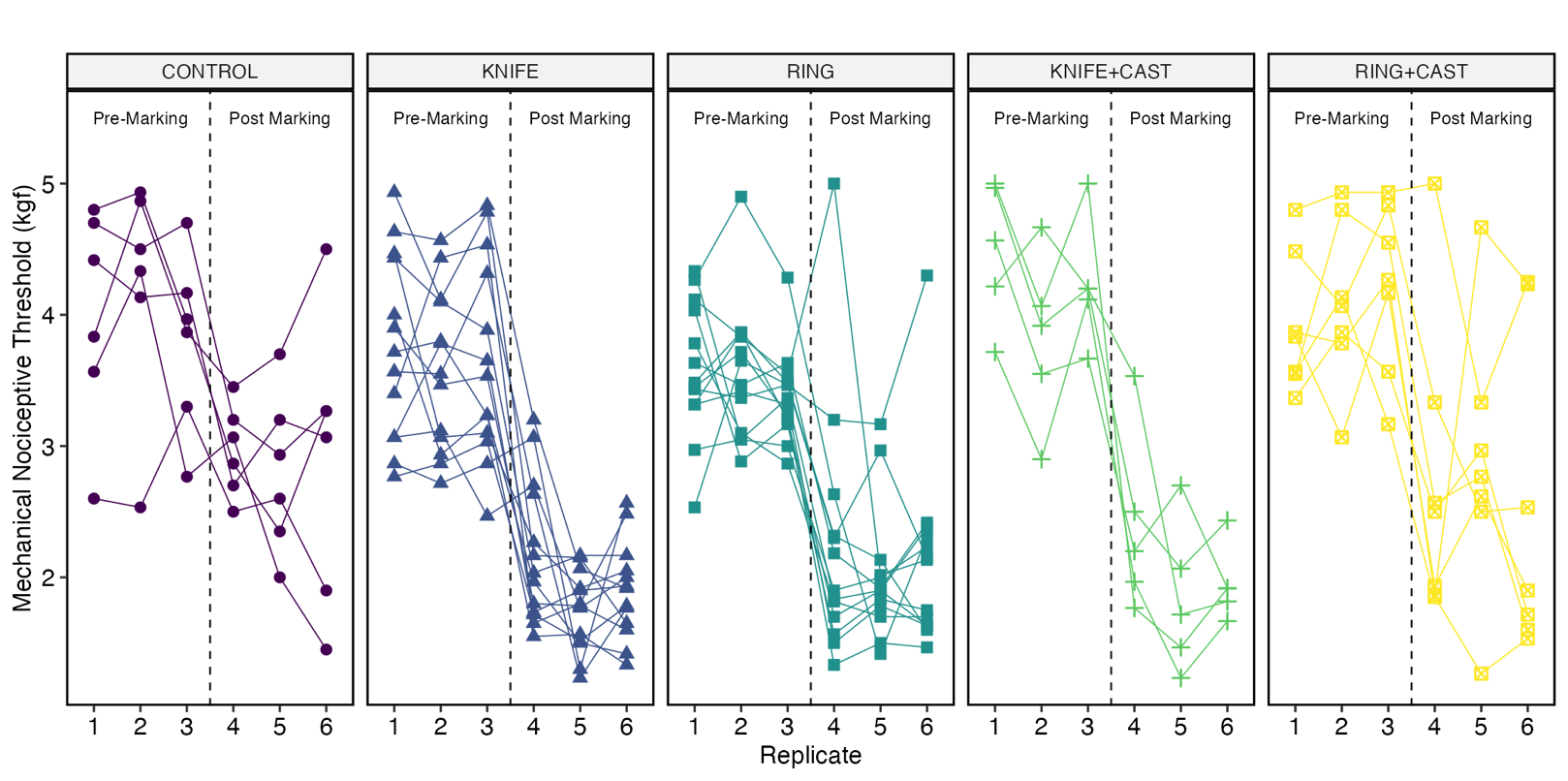


**Figure S.1 |** Mechanical nociceptive threshold at the base of the tail in lambs across replicate measurements pre- (replicates 1, 2, and 3) and post-marking (replicates 4, 5, and 6). Each facet shows lambs within a marking group; either Control (sham handling), Knife (hot knife tail docked), Ring (ring tail docked), Knife+Cast (hot knife tail docked and ring castrated), Ring+Cast (ring tail docked and castrated).


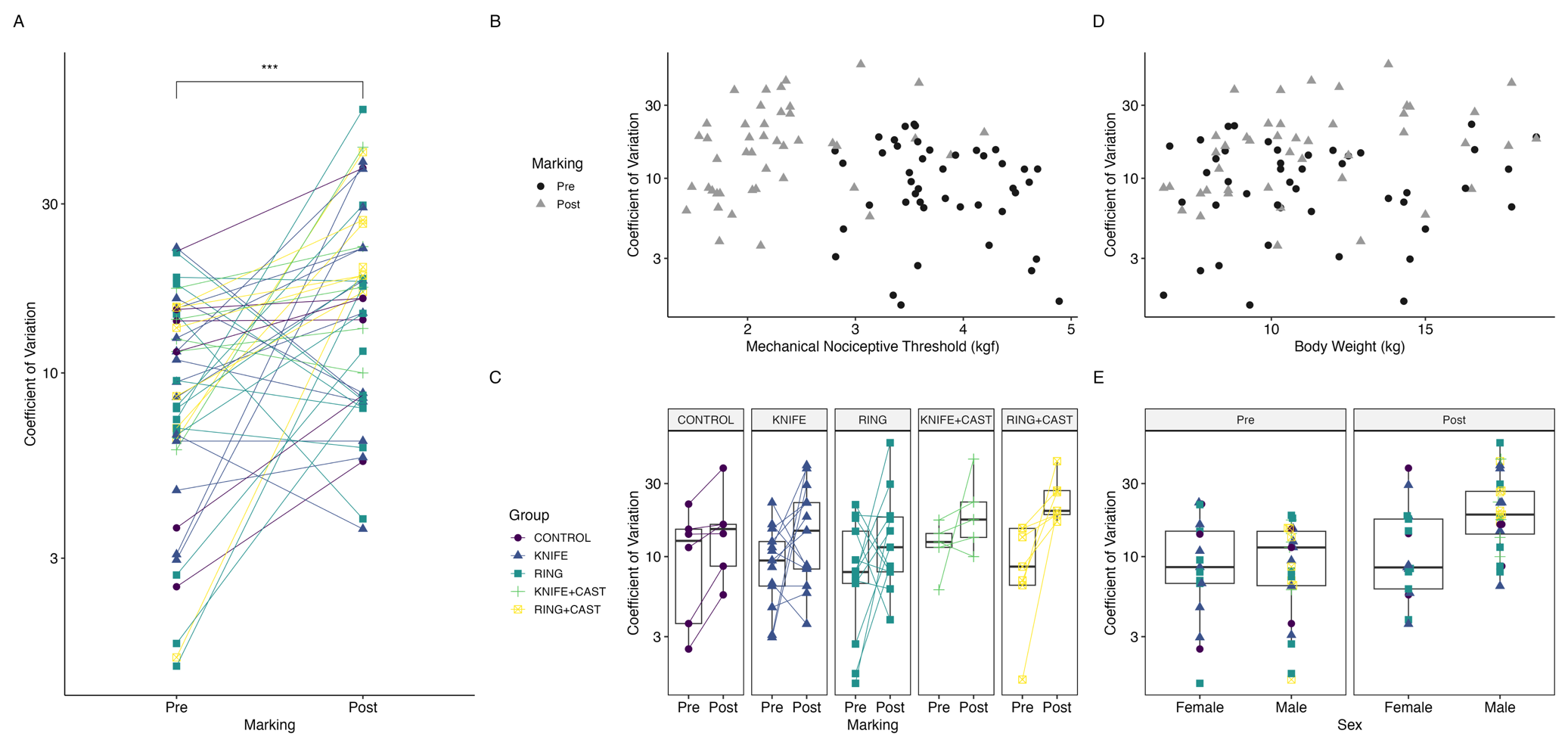


**Figure S.2 |** Relationship between within-animal/-occasion (pre- or post-marking) coefficient of variation (CV) and marking status (A), mean within-animal/-occasion (pre- or post-marking) mechanical nociceptive threshold (MNT) colored by marking status (pre- and post-) (B), marking status by marking group (C), body weight colored by marking status (pre- and post- )(D), and sex faceted by marking status (E). Data are displayed on a log10 scale.


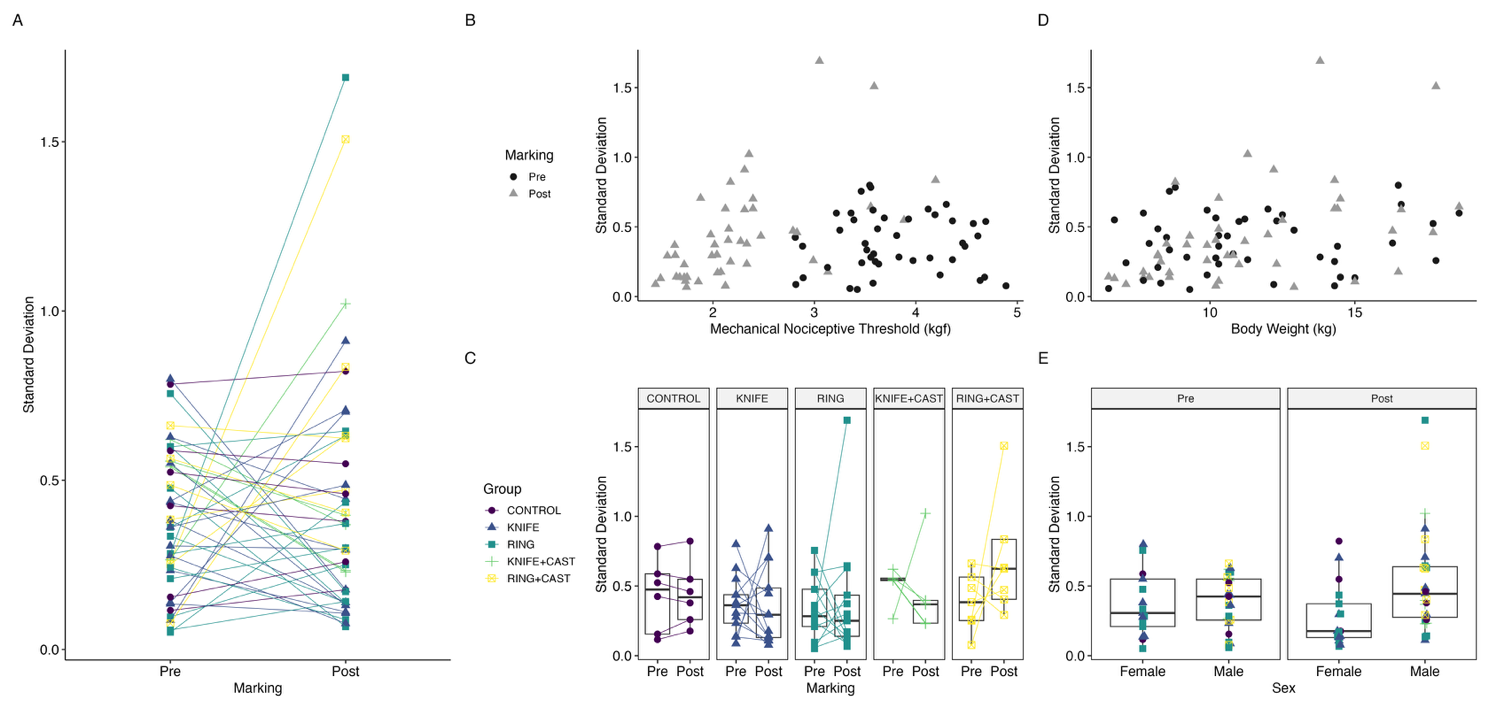


Figure S.3.1 | Relationship between within-animal/-occasion (pre- or post-marking) standard deviation (SD), including two outliers, and marking status (A), mechanical nociceptive threshold (MNT) colored by marking status (pre- and post- ) (B), marking status by marking group (C), body weight colored by marking status (pre- and post- ) (D), and sex faceted by marking status (E).


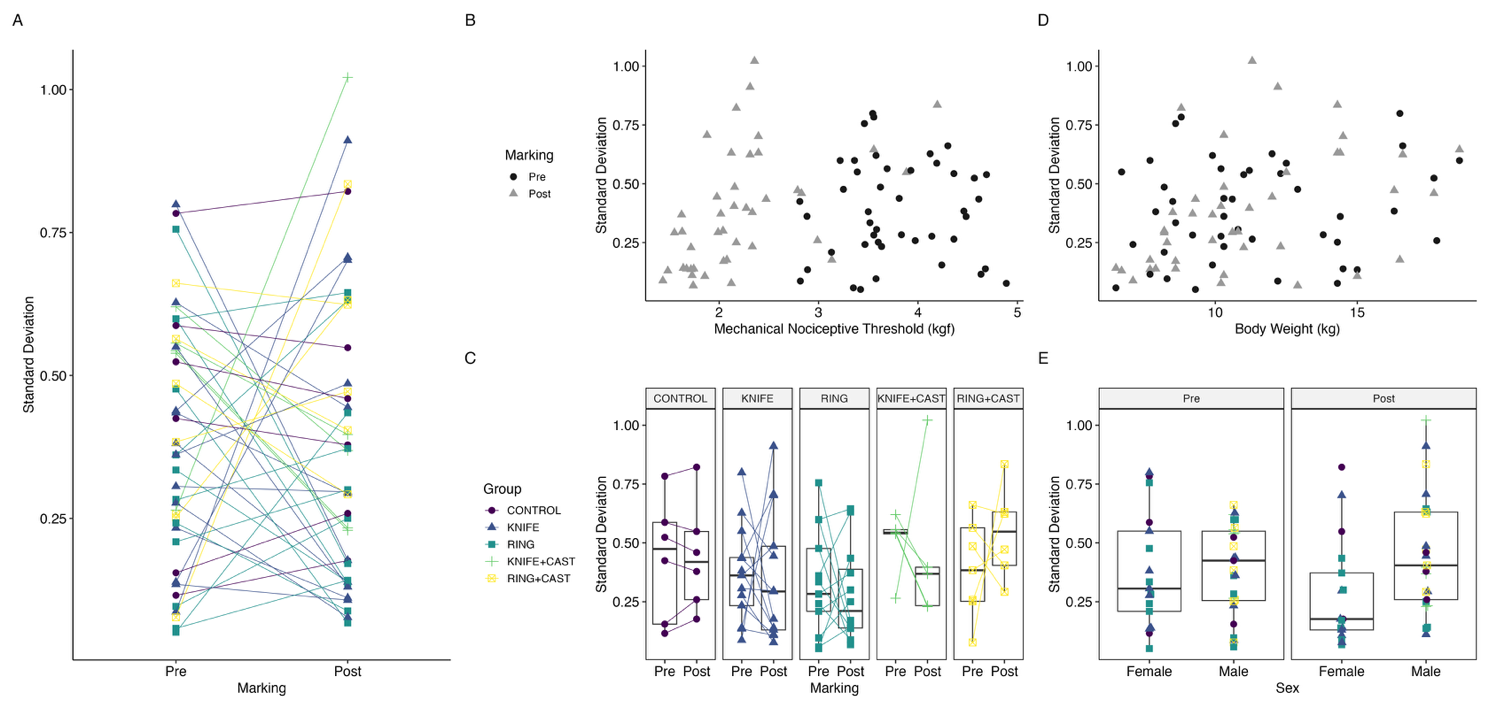


**Figure S.3.2 |** Relationship between within-animal/-occasion (pre- or post-marking) standard deviation (SD), excluding two outliers, and marking status (A), mechanical nociceptive threshold (MNT) colored by marking status (pre- and post- ) (B), marking status by marking group (C), body weight colored by marking status (pre- and post- ) (D), and sex faceted by marking status (E).
